# Understanding motives for illicit medicinal cannabis use: an exploratory analysis in a medical cannabis program

**DOI:** 10.1186/s42238-025-00284-w

**Published:** 2025-07-18

**Authors:** Carter Reeves, Lirit Franks, A. Taylor Kelley, Michael Incze, Adam J. Gordon, Ziji Yu, Eden Flake, Gerald Cochran

**Affiliations:** 1 Program for Addiction Research, Clinical Care, Knowledge and Advocacy (PARCKA), Division of Epidemiology, Department of Internal Medicine, University of Utah School of Medicine, Salt Lake City, UT USA; 2 Division of General Internal Medicine, Department of Internal Medicine, University of Utah School of Medicine, Salt Lake City, UT USA; 3 Informatics, Decision-Enhancement, and Analytic Sciences (IDEAS) Center, VA Salt Lake City Health Care System, Salt Lake City, UT USA

**Keywords:** Medical Cannabis, Marijuana, Illicit use, Barriers, Contributors, Motives

## Abstract

**Background:**

Medical Cannabis (MC) is authorized in numerous state-legislated programs to treat approved medical conditions. Notwithstanding MC access, some participants continue to use cannabis purchased outside of a state licensed MC pharmacy, otherwise known as illicit medicinal cannabis (IMC), to treat their medical conditions. Identifying barriers and contributors to MC use and motives for IMC use can promote safety, improve program design, and inform future research efforts.

**Methods:**

This exploratory analysis utilized baseline survey data from a convenience sample-based prospective cohort evaluation of newly registered (< 6 months) adult participants in Utah’s MC program who had been diagnosed with chronic pain, post-traumatic stress disorder, and/or cancer. Participants completed surveys assessing physical and mental health, program experience, and barriers and contributors to MC access. We employed descriptive analysis, chi-squared analysis, and logistic regression to identify factors influencing IMC use.

**Results:**

Among 273 MC program participants screened for eligibility, 227 were enrolled in the cohort evaluation, and 211 participants completed the baseline survey. Approximately 1 in 10 survey respondents (*N* = 24, 11.9%) reported IMC use within the past two weeks. Participants accessing IMC were 40.5 years old, 58.3% male, 70.8% employed, and 87.5% white. Participants using IMC reported barriers to MC, including product cost (*n* = 19, 79%) and assurance of adequate supply (*n* = 11, 45.8%) as the most common motives for IMC use. Participants who reported experiencing MC access barriers were significantly more likely to report IMC use than those reporting no barriers (Odds Ratio (OR) = 4.73, *p* <.001). Participants using IMC reported lower levels of trust in (*p* <.04) and reliance (*p* <.02) upon the state program and less reliance on MC pharmacists (p’s < 0.01). However, participants who relied on the state program for MC information were less likely to report IMC use (Adjusted Odds Ratio AOR = 0.16, *p* <.05).

**Conclusions:**

In a state MC program, barriers related to MC access and cost indicated a significant increase in the likelihood of IMC use, while reliance on the state program for MC information indicated a significant decrease in the likelihood of IMC use. Future research can explore how increasing affordable access to MC and availability of reliable information may affect IMC use.

## Introduction

Medical Cannabis (MC) is an increasingly common treatment for a variety of medical conditions. Over four million Americans across 38 states and the District of Colombia are registered with their state MC programs [1]. Individual states and districts have taken responsibility to legislate MC use and distribution for their constituents due to the current Drug Enforcement Administration Schedule I status of cannabis.

Despite MC being legally available in most states, cannabis remains the most used illicit substance in the United States [2]. Previous research has demonstrated that some patients authorized to purchase MC still utilize illicit cannabis to treat their health conditions that qualify them for the MC program, with prevalence of illicit use as high as 10% [3]. This notable prevalence of illicit cannabis use, cannabis purchased outside of a state licensed MC pharmacy, or illicit medicinal cannabis (IMC), raises concern when considering its potential health and legal consequences. Engaging in illicit cannabis use, regardless of motive, can result in legal repercussions such as incarceration [4], undesired or unpredictable side effects from illicit products with higher general THC content [5], and a lack of reliable product information, quality, standardization, or access [6] - which all may impact treatment efficacy for participants’ medical conditions [7]. Despite the potential negative consequences to IMC use, MC program participants still access IMC at notable rates.

IMC use might be explained in part by potential barriers to MC within state programs. For example, previous research suggests experiencing healthcare-related barriers may increase illicit substance use [8], with one study reporting that 24.7% of participants using illicit substances experience unmet healthcare needs [9]. These data suggest similar concerns may exist in MC programs.

Notwithstanding barriers to MC access, medical provider and pharmacist relationships may also influence IMC use. For example, positive provider relationships and trust have been shown to attenuate illicit substance use [10, 11]. Pharmacists can also facilitate substance use treatment through screening, intervention, and referrals [12]. Since healthcare providers are involved in MC participants’ enrollment and treatment in MC state programs, they can have a substantive impact on IMC use. Medical professionals can leverage expertise and trusting relationships with participants to recommend safer practices around cannabis use as well as alternative low-cost treatment options for chronic health conditions.

These risks related to IMC use and the limited available empirical information available in the field—especially related to MC program-level factors [13] — underscore the need for further research into the motivations behind this behavior. Understanding why MC program participants engage in IMC use may help identify ways to reduce overall illicit cannabis use, limit health disparities, and enhance the health and safety of individuals in MC programs. This exploratory study aimed to investigate barriers and contributors to MC access and the motivations of MC program participants who use IMC.

## Methods

### Design, data source, and participants

We conducted cross-sectional exploratory analyses of self-reported baseline variables from the Study of Utahns’ Beliefs and Life Experiences with Integrative Medicine (SUBLIME). This program evaluation aimed to measure participants’ reported benefit and efficacy of the MC program by assessing many factors, including variables measuring and influencing IMC use in the state of Utah. SUBLIME was a mixed-methods prospective cohort evaluation of Utah’s Medical Cannabis Program ongoing from 2023 to 2025. Notably, Utah has a medical-only cannabis program and adult use of cannabis is prohibited. Evaluation procedures were approved by the University of Utah Institutional Review Board.

The SUBLIME MC cohort included adult (*≥* 18 years old) Utah residents who were state-registered MC program participants for ≤ 6 months. Participants were English-speaking who were able to provide reliable contact information for themselves and two other collateral contacts. Additionally, we restricted cohort enrollment to diagnoses of one or more of the three most prevalent qualifying conditions in the state program to obtain a generalizable sample: chronic pain, post-traumatic stress disorder, or current cancer treatment. Diagnoses were self-reported and not mutually exclusive. Exclusion criteria included current incarceration, pregnancy, and/or mania or psychosis within the past month to protect vulnerable populations and increase data accuracy.

To recruit cohort participants, we distributed recruitment material at MC pharmacies, advertised on social media platforms (Facebook, Twitter, LinkedIn, Instagram), and attached recruitment material in the Utah Department of Health and Human Services (DHHS) Center for Medical Cannabis monthly newsletter. Our team contacted eligible prospective participants to provide details regarding evaluation participation, and for those eligible and interested, to request written informed consent to participate. After enrollment, a link to an online survey was emailed to participants at three time points: baseline, 6-months post-enrollment, and 12-months post-enrollment. Study data were collected and managed using Research Electronic Data Capture (REDCap), a secure web-based application.

### Assessments

We assessed baseline survey data regarding physical health, mental health, and MC program experience using multiple survey instruments that specifically assessed potential barriers and potential contributors to IMC use: (1) the Cannabis as Medicine Survey (CAMS) and (2) Patient Experiences Survey.

#### Cannabis use

The CAMS is a common measure to assess participant cannabis usage [14, 15]. The CAMS consisted of questions assessing the age of first access to cannabis, weekly medicinal cannabis cost, prescriber qualifications, pharmacy, reasons for discontinuing MC, barriers to MC access, and IMC use. We specifically employed participant responses to the MC access items in our current analysis as previous research has indicated that healthcare-related barriers may increase the risk of illicit substance use [8, 9]. Participants were asked, “Did you ever have problems getting medicinal cannabis?”. If participants indicated that they did, they were prompted to choose from a set of barriers relating to healthcare provider access, program enrollment process, MC product access, and individual factors largely related to perception and cost (See Appendix A).

#### Trust and reliance

The Patient Experiences Survey assessed program satisfaction factors such as distance to the pharmacy, referral source, program application, pharmacy experience, product variety, program accessibility, program efficacy, and varying MC information sources. The survey also asked participants to report on levels of trust and reliance on MC information sources. Trust and reliance on various sources of MC information have been previously identified as potential contributors that may influence IMC use [10–12]. Trust items asked participants to rate how much they trusted people/entities to address questions/concerns about MC use on a 5-point Likert scale from “Strongly distrust” to “Strongly trust”. Reliance items asked participants to report where they receive their MC information from amongst the included entities. Responses included “No to little information,” “Some information,” or “Majority of information.” Trust refers to participants indicating that they either trust or strongly trust the information source and reliance refers to where participants indicated they receive the majority of their information regarding MC.

#### Demographics

In addition, demographic information including age, sex, employment status, qualifying medical condition, educational background, marital status, ethnicity, and race were collected.

### Analyses

This exploratory analysis was designed to be hypothesis-generating in nature. Analysis included utilizing descriptive statistics to characterize the analytical cohort. The “IMC use” group included participants who indicated IMC use at least once in the previous 12 months, with plans to continue in the future. The “no IMC use” group included participants who indicated no IMC use in the previous 12 months with no plans for future use or no IMC lifetime use. Participants who used IMC more than 12 months ago were also included in the “no IMC use” group. We described survey responses for “IMC use” and “no IMC use” participants using measures of motivation and behavior for IMC use gathered in the survey.

Chi-squared analysis was employed to determine if there were any group differences among reported barriers/contributors to MC access. Fisher’s exact test was used to compensate for cell counts less than five. Univariate logistic regression was then used to identify associations between barriers/contributors and current IMC use, using *p* <.10 as the statistical significance cutoff. This cutoff was chosen to aid the exploratory nature of the project analysis. Once possibly associated factors were identified, a stepwise multivariate logistic regression was employed to identify important risk factors that were associated with an influence in IMC use. In the final stepwise model, factors were considered statistically significant and retained if *p* <.05. All data cleaning and statistical analysis were conducted using STATA 18.

## Results

### IMC participants characteristics

**Fig. 1:**
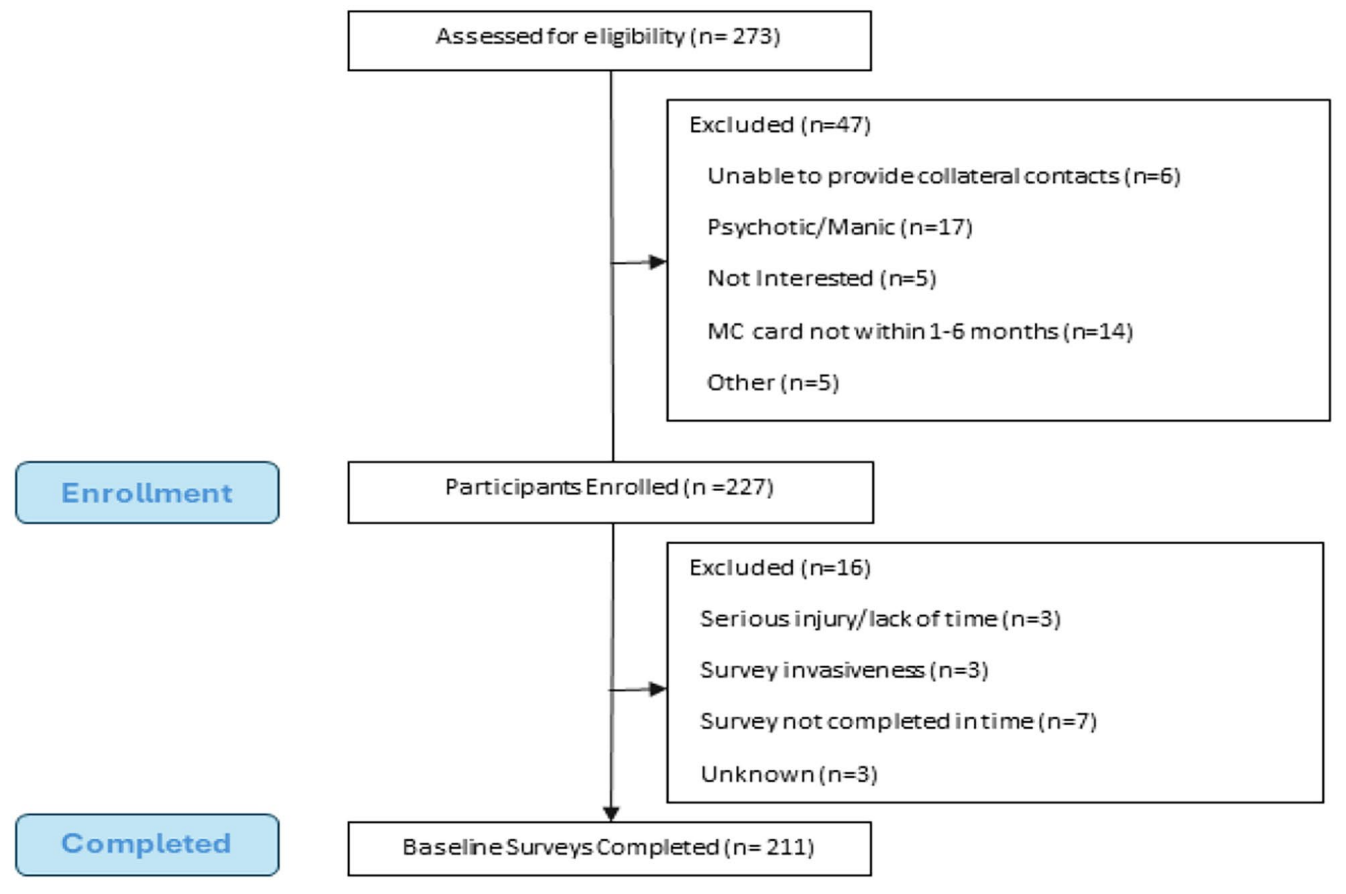
STROBE Chart

Among 273 MC program participants screened, 47 also met exclusion criteria, leaving 227 enrolled in the evaluation cohort. There were 16 enrolled participants who did not complete the survey, primarily because they did not complete the survey within the given timeframe (*n* = 7). 211 participants completed the baseline survey (Fig. 1). In our sample, 11.9% (*n* = 24) of participants reported IMC use. These participants were mostly white (83.3%) and male (58.3%). and slightly older (40.5 vs. 38.7 years of age) than those who did not report IMC use. Chronic pain condition is reported higher in participants indicating IMC use than those not reporting IMC use (87.5% vs. 75.9%). There were no differences between groups for any demographic variables (P’s > 0.05; Table 1).

**Table 1:** Demographics of patients with and without IMC use

Baseline Characteristic	IMC Use (*n* = 24)		No IMC Use (*n* = 187)	
	$\overline x$	*SD*	$\overline x$	*SD*
Age	40.5	2.51	38.7	0.86
	*n*	*%*	*n*	*%*
Male	14	58.3	73	40.1
Non-Hispanic/Latino	21	87.5	156	85.7
White	21	87.5	154	84.6
Chronic Pain*	21	87.5	142	75.9
PTSD*	7	29.2	62	33.2
Cancer*	0	0.0	11	5.9
> High School Educ.	20	83.3	149	79.7
Married	12	50.0	74	39.6
Employed	17	70.8	112	61.5

*Note: P’s > 0.05 for comparisons between IMC and No IMC for all characteristics.*

*Qualifying conditions were not mutually exclusive.

### Barriers

Participants accessing IMC reported problems getting MC at a greater rate than those who did not access IMC (54.2% vs. 20.2%, *p* =.002; Table 2). There were specific barriers that were experienced at higher rates among participants with IMC use versus participants without IMC use. These statistically significant barriers (p’s < 0.10) were categorized into three groups based on observed commonalities: (1) healthcare access, (2) program enrollment, and (3) perception of MC use.

Regarding healthcare access (1), participants using IMC reported a greater proportion of lack of health insurance to access MC (16.7% vs. 3.9%, *p* =.03) and greater problems with the cost of MC products (45.8% vs. 15.4%, *p* <.001). Concerning experiences with program enrollment (2), participants using IMC reported greater problems compared to those without IMC with renewal requirements (25.0% vs. 0.6%, *p* =.04), enrollment costs (29.2% vs. 7.1%, *p* =.003), the renewal process (16.7% vs. 4.4%, *p* =.04), and confidence to obtain MC products (12.5% vs. 1.7%, *p* =.02), respectively. Regarding perceptions of MC use (3), participants using IMC reported higher rates compared to those without IMC of provider stigma (8.3% vs. 0.6%, *p* =.04) and fear of discrimination at work (37.5% vs. 8.8%, *p* =.001).

**Table 2:** Chi-Squared analysis of contributors and barriers to illicit medical Cannabis use

Variables	IMC Use		No IMC Use		*p*	Χ²
	n	%	n	%		
*Did you ever have problems getting medicinal cannabis?	13	54.2	37	20.2	0.002	13.3
**Barriers**						
**Healthcare Provider Access**						
^+^Incompatible insurance	0	0.0	1	0.6	0.88	0.13
*^+^Lack of insurance coverage	4	16.7	7	3.9	0.03	6.9
^+^Long wait time	2	8.3	5	2.8	0.19	2.0
^+^No MC-authorized provider available	0	0.0	6	3.3	0.47	0.81
**Program Enrollment Process**						
^+^Difficulty navigating state website	3	12.5	7	3.9	0.10	3.4
^*+^Difficulty in knowledge of renewal requirements	6	25.0	1	0.6	< 0.001	38.6
*^+^Problems with renewal process	4	16.7	8	4.4	0.04	5.8
*Problems with enrollment cost	7	29.2	6	7.1	0.003	11.7
**Medical Cannabis Accessibility**						
*^+^Provider Stigma/Bias	2	8.3	1	0.6	0.04	9.0
^+^No medical cannabis near them	3	12.5	5	2.8	0.05	5.4
^+^Incompatible pharmacy hours	2	8.3	5	2.8	0.19	2.0
**Individual Factors**						
*Cost of MC products	11	45.8	27	15.4	0.001	12.8
^+^Transportation issues	3	12.5	10	5.5	0.18	1.8
^+^Caregiver responsibilities	1	4.2	0	0.0	0.12	7.6
^+^Not safe to keep cannabis products in my home	1	4.2	2	1.1	0.31	1.4
*Fear of discrimination at work	9	37.5	16	8.8	0.001	16.4
*^+^Lack of confidence to obtain MC products	3	12.5	3	1.7	0.02	8.8
Negative cultural perception of MC	7	29.2	10	5.5	0.001	15.7
Negative friend/family perception	5	20.8	14	7.69	0.05	4.4
**Contributors to IMC Use**						
**Trusted Sources of MC Information**						
Trust in MC Pharmacist	20	87.0	164	89.1	0.24	1.13
Trust in Medical Provider	21	87.5	156	89.1	0.51	06
*Trust in State MC Program	10	45.5	116	67.4	0.04	4.14
Trust in Friends	17	73.9	116	67.8	0.37	0.35
Trust in Internet	9	37.5	55	31.8	0.37	0.31
**Reliance on Sources of MC Information**						
*Info Reliance on MC Pharmacist	8	33.3	103	58.9	0.01	10.6
Info Reliance on Medical Provider	18	75.0	155	87.6	0.09	2.79
*Info Reliance on State MC Program	9	37.5	111	63.1	0.02	5.75
Info Reliance on Friends	19	79.2	141	79.7	0.57	0.003
Info Reliance on Internet	19	79.2	138	78.0	0.57	0.018

*Significant difference (p < *.05).*^*+*^*Measured using Fisher’s Exact Test.*

Results similarly showed that experiencing barriers to MC access was significantly associated with an increase in the likelihood of IMC use (See Table 3). When explicitly asked in the survey, experiencing problems getting MC was associated with an increased likelihood of IMC nearly fivefold (Odds Ratio OR = 4.73, *p* =.001). For healthcare access (1), experiencing lack of insurance (OR = 4.86, *p* =.02), no MC access through pharmacies nearby (OR = 4.91, *p* =.04), or high cost of MC products (OR = 4.91, *p* =.001) were all associated with increased odds of IMC use. For program enrollment (2), increased odds of IMC use were associated with experiencing problems with state website navigation (OR = 3.47, *p* =.09), renewal requirements (OR = 58.7, *p* =.001), the renewal process (OR = 4.23, *p* =.023), enrollment costs (OR = 5.66, *p* =.001), and confidence to obtain MC products (OR = 12.5, *p* =.01). Regarding perception of MC use (3), experiencing problems with provider stigma (OR = 16.0 *p* =.026), fear of discrimination at work (OR = 6.99, *p* =.001), cultural perception (OR = 7.69, *p* =.001), and friends or family’s perception (OR = 3.32, *p* =.04) were associated with increased odds of using IMC.

**Table 3:** Univariate logistic regression of barriers and contributors to MC impact on IMC use

Variables	OR	SE	*p*	95% CI
*Did you ever have problems getting medicinal cannabis?	4.73	2.13	0.001	[1.95, 11.4]
**Barriers**				
**Healthcare Provider Access**				
*Lack of insurance coverage	4.86	3.25	0.02	[1.31, 18.1]
Long wait time	3.12	2.71	0.19	[0.57, 17.1]
**Program Enrollment Process**				
*Difficulty navigating state website	3.47	2.52	0.09	[0.83, 14.4]
*Difficulty in knowledge of renewal requirements	58.7	65.0	< 0.001	[6.69, 514.8]
*Problems with renewal process	4.23	2.20	0.03	[1.17, 15.3]
*Problems with enrollment cost	5.66	3.05	0.001	[1.97, 16.3]
**Medical Cannabis Accessibility**				
*Provider Stigma/Bias	16.0	19.9	0.03	[1.39, 183.8]
*No medical cannabis near them	4.91	3.76	0.04	[1.10, 22.1]
Incompatible pharmacy hours	3.93	3.52	0.13	[0.68, 22.7]
**Individual Factors**				
*Cost of MC products	4.91	2.27	0.001	[1.99, 12.1]
Transportation issues	2.67	0.188	0.17	[0.67, 10.6]
Not safe to keep MC products in home	3.8	4.74	0.28	[0.33, 43.6]
*Fear of discrimination at work	6.99	3.53	< 0.001	[2.59, 18.8]
*Lack of confidence to obtain MC products	12.5	11.8	0.01	[1.97, 79.1]
*Negative cultural perception of MC	7.69	4.34	< 0.001	[2.54, 23.2]
*Negative friend/family perception	3.32	1.92	0.04	[1.07, 10.3]
**Contributors to IMC Use**				
**Trusted Sources of MC Information**				
Trust in MC Pharmacist	0.49	0.34	0.30	[0.13, 1.88]
Trust in Medical Provider	0.85	0.57	0.81	[0.23, 3.13]
*Trust in State MC Program	0.40	0.18	0.05	[0.16, 0.99]
Trust in Friends	1.34	0.67	0.56	[0.50, 3.60]
Trust in Internet	1.29	0.58	0.58	[0.53, 3.12]
**Reliance on Sources of MC Information**				
*Info Reliance on MC Pharmacist	0.35	0.16	0.02	[0.14, 0.86]
Info Reliance on Medical Provider	2.35	1.23	0.10	[0.84, 6.55]
*Info Reliance on State MC Program	0.43	0.16	0.02	[0.21, 0.88]
Info Reliance on Friends	0.97	0.52	0.96	[0.34, 2.78]
Info Reliance on Internet	1.07	0.57	0.89	[0.38, 3.06]
**Motives to IMC Use**				
*Ensure an Adequate Supply	14.1	7.39	< 0.001	[5.01, 39.4]
*Cost of Products	17.9	9.67	< 0.001	[6.21, 51.6]
Difficulty obtaining a prescription	3.93	3.52	0.13	[0.68, 22.7]
*Cost of consultation	4.07	3.03	0.06	[0.95, 17.5]
*Improve effectiveness	35.2	40.2	0.002	[3.74, 330.5]

*Significant Difference (p < *.10). NOTE: Variables with ≤ 1 response in any condition were not included in the analysis due to collinearity and subsequentially not included in the table.*

### Potential contributors to IMC use

Participants using IMC reported differing levels of trust and information reliance than those not using IMC. These participants reported significantly lower levels of trust in the state MC Program (45.5% vs. 67.4%, *p* =.04). Participants using IMC reported lower levels of information reliance as compared to those not using IMC on two sources: MC pharmacists (33.3% vs. 58.9%, *p* =.01) and the state MC program (37.5% vs. 63.1%, *p* =.02). Participants from both groups reported similar levels of trust and reliance among the other sources of information (See Table 2).

Sources of trust and information reliance correlated with IMC use. Trusting and relying on the state MC program for information indicated a decrease in the odds of IMC use (OR = 0.43, *p* =.02). Relying on information from the MC pharmacists (OR = 0.35, *p* =.02) and the state program (OR = 0.43, *p* =.02) were similarly associated with lower odds of IMC use.

### Motives to IMC use

**Fig. 2:**
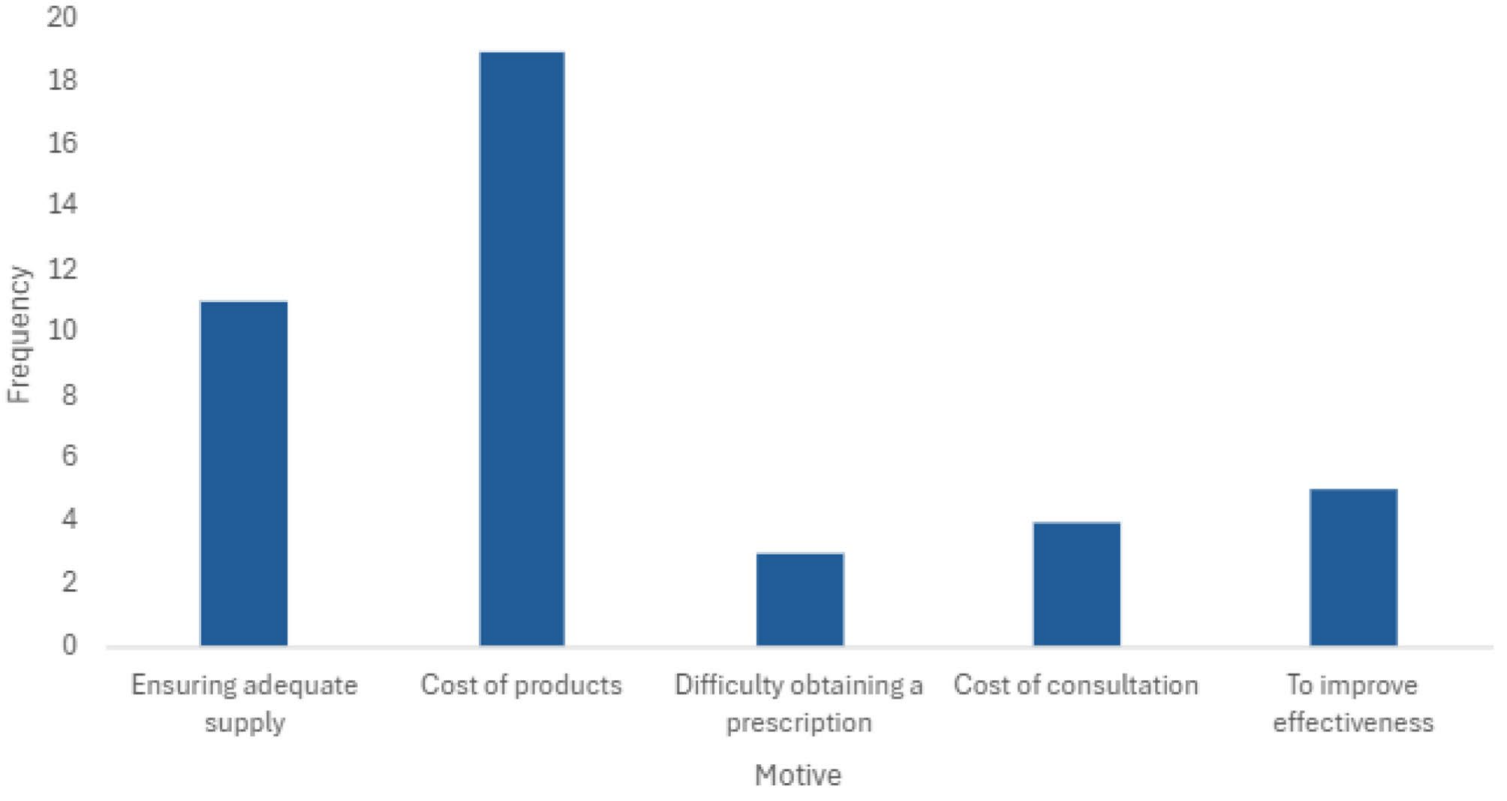
Reasons for IMC Use Among Participants with IMC Use

The most cited reason for using IMC indicated by respondents was the cost of products (*n* = 19, 79%) and difficulty obtaining a prescription was the least common reason (*n* = 3, 8.3%, See Fig. 2). Several identified participant-reported motives increased odds of IMC use including ensuring an adequate supply (OR = 14.1, *p* =.001), cost of MC products (OR = 17.9, *p* =.001), and improving effectiveness (OR = 35.2, *p* =.002).

We conducted a multivariate logistic regression, including all relevant factors identified from the univariate analysis in the full model, and conducted stepwise variable selection. The final model included three risk factors that potentially increased the risk of IMC use (See Table 4). Both concerns about accessing an adequate supply of MC (Adjusted Odds Ratio (AOR) = 6.96, *p* =.004) and concerns about the cost of MC products (AOR = 20.1, *p* <.001) were significantly associated with higher odds of IMC use, while relying on information from the state MC program was associated with lower odds of IMC use (AOR = 0.16, *p* =.05).

**Table 4:** Stepwise regression of barriers and contributors to illicit medicinal Cannabis use

Barriers and Contributors	AOR	SE	*p*	95% CI
Ensuring an adequate supply of MC	6.96	4.70	0.004	[1.85, 26.1]
Cost of MC Products	20.1	13.96	< 0.001	[5.16, 78.4]
Info Reliance on State MC Program	0.16	0.15	0.049	[0.03, 0.99]

*Note: Only significant factors (p* < *.05) were kept in the final model. All surveyed demographic variables were included in the model but proved to be non-significant.*

## Discussion

IMC use is a prevalent behavior among MC program participants, with these data demonstrating that one in ten MC patients use IMC [3]. In order to address this potentially dangerous behavior, it is critical to understand the elements that contribute to IMC use. This exploratory analysis revealed factors that may influence IMC use. Our analysis focused on barriers and contributors to accessing MC, the impact of sources of information related to IMC use, and specific motivations for IMC use.

### Barriers

***Cost***. Experiencing problems with MC product cost was shown as a main motive for IMC use, as 79.2% of participants using IMC reported this barrier. This finding remains consistent among MC participants in other programs across the country, as participants often report that the cost of products was either an issue or unaffordable [16–18]. However, lowering the cost of MC may not address these product issues. Previous research showed that while price influences product choices, demand is relatively inelastic [19], indicating that decreasing the price of MC may not lead to lower IMC use. Nonetheless, due to the impact of product price on IMC use, cost may still be a relevant issue for MC participants who use IMC.

#### Access

The increase in IMC use was most highly associated with barriers relating to healthcare access, program enrollment experiences, and perception of MC use. Frequently reported access barriers, including problems with insurance or no MC pharmacies nearby, may indicate that many MC participants using IMC resort to these illicit products due to relative unavailability of legal MC options. Thus, future studies to assess whether increasing access to MC programs might reduce IMC use may be warranted. However, facilitating broader access to cannabis through MC programs may increase the risk of negative health effects for some individuals, especially at-risk groups such as cannabis-naive patients, older adults [20] and adolescents [21] due to the risk of unfamiliarity and long-term health consequences specific to these populations. Policies that seek to increase MC access may need to be accompanied by research measuring both the benefits and collateral harms of increasing legal access to cannabis. It is worth noting that patients who reside in states with only MC options may face different barriers than patients in states with both medical and adult-use options. Patients in states with both programs may be less incentivized to access MC because adult-use options may be cheaper or easier to access.

#### Program participation

Experiences within program enrollment or card renewal were also found to increase the likelihood of IMC use. Our results suggested participants accessing IMC may be deterred from MC use due to program-specific issues such as understanding renewal requirements, navigating the state website, and the enrollment cost. Participants in other MC programs have reported similar issues [16–18]. Previous research into factors that influence switching from illicit to licit has shown that similar issues such as price, regulation, and reduced accessibility act as barriers to MC use [22]. Our sample presents similar trends while also providing further understanding of specific barrier effects and protective factors against IMC use.

#### Stigma

Our analysis also indicated that participants’ and providers’ perceptions of MC use significantly affected IMC use. Although our results showed that participants who use IMC experienced negative cultural or familial perception of MC use at similar rates as those who did not use IMC, participants who use IMC were more influenced by these negative perceptions as fear of experiencing discrimination at work or stigma by MC providers was associated with increased IMC use. Although public sentiments about MC use across the country are generally positive [23], if a participant is internalizing negative perceptions of use, they might forgo public purchasing, typical at a pharmacy, to avoid these negative perceptions [24]. 

Additionally, previous research into motives for IMC use in treating medical conditions has found that healthcare provider perceived stigma motivates IMC use [25]. Our analysis produced similar results as provider stigma was shown to be associated with increased IMC use. People who use drugs are often stigmatized by the healthcare providers treating them [26], which can preclude discussions about the relative risks and benefits of MC with their medical providers. Instead, some participants may opt to use IMC to avoid negative public perception, shame, or stigma. Educational and stigma-reducing campaigns for healthcare providers may prove beneficial to reduce stigma surrounding MC use, thereby possibly reducing IMC use and improving care for program patients.

There were a few factors presented as barriers in the survey that did not influence the likelihood of IMC use. Problems related to pharmacy customer service such as long wait times, or incompatible hours did not affect IMC use in our sample of participants. Individual factors such as transportation or home storage issues were similarly not significant in our sample. It is possible that, despite encountering these challenges, participants may not perceive the associated barriers as sufficient to instigate use of IMC.

### Potential contributors

#### Program information

We also analyzed whether the degree to which participants relied on, or trusted different sources of information was associated with IMC use. We found that relying on information from both MC pharmacists and state MC program resources was associated with a decrease in IMC use. If participants trust and receive information from these sources, thereby possibly strengthening their connection and reliance on the MC program, they would be more likely to access legal products than IMC [27]. Connection and reliance on the program may be achieved through efficacious care and positive interactions.

Notably, trust in MC pharmacists did not have a significant association with IMC use while reliance did, suggesting that information reliance may have some greater influence on behavior than trust. Participants in MC programs often rely on information from a variety of sources including the internet, friends, and family [28]. As information about cannabis is often ambiguous and illusive [29], it is reasonable to assume that MC patients will continue to favor availability over creditability. As a result, supporting the collection and dissemination of valid and reliable cannabis research may provide a reduction in IMC use.

Our final analytical model highlighted three significant influences: (1) Ensuring an adequate supply and (2) cost of MC products was associated with an increased likelihood of IMC use while (3) information reliance on state resources was correlated with decreased IMC use. Based on these results, we hypothesize that low availability and high cost of MC products may increase IMC use while reliance on state resources may attenuate use. Future studies could focus efforts on measuring patterns of MC and IMC use when MC costs are reduced or measure how cannabis knowledge affects MC adherence.

### Limitations

There are limitations to the study findings that should be considered. The exploratory nature of this study and smaller size of the current IMC group may limit the generalizability of the findings, signaling the necessity of further research into this topic. The patient-reported data also represents a small cohort in a single state, which may limit external validity. Although our sample possessed important points of possible representativeness of the overall state MC population with regards to condition, it may not be representative of other MC populations across the US or other parts of the world where cannabis use is legal, as barriers and potential contributors may differ. Moreover, further research should systematically consider illicit MC use in other jurisdictions, both within and outside the US, as well as among individuals who have not attempted to access the legal system, to better understand how these barriers and factors influence usage. Further studies should also examine how perceptions of effectiveness and quality affect IMC use.

Evaluation cohorts were also created using a convenience sampling method, which could introduce potential bias to the sample. Future studies may consider probability sampling methods to control for these threats. This analysis was conducted with baseline survey data that may not accurately capture the lasting perceptions and demographics of those in the program, as these factors may shift as the program continues to grow. Additionally, the frequency of IMC use in our sample may be underrepresented due to social desirability bias. Parts of our analysis relied on variables that contained cell sizes with less than five responses. We used Fisher’s exact test as a method to compensate for small cell sizes. We also observed large confidence intervals in some of our regression analyses due to our small size. Future studies should consider larger samples to correct these errors. Additionally, correction for multiple comparisons was not undertaken for this exploratory analysis. Larger confirmatory studies should utilize such corrections to better understand the impact of these identified factors on IMC use [30]. 

## Conclusion

As state MC programs develop, the prevalence and clinical implications of IMC use must be considered. In this exploratory study, we identified key factors that may influence the likelihood of use IMC among participants enrolled in a MC program. Our findings suggest that barriers related to MC supply and cost may contribute to IMC use, while reliance on state program information appears to protect against this behavior. Additional factors, such as enrollment barriers and public perception may be influential in participants’ decision-making processes. While this study offers valuable insights, more comprehensive research based on these preliminary findings is needed to develop effective strategies to mitigate IMC use and promote safe MC program participation.

## Appendix A

**Table A:** List of surveyed MC barriers

Surveyed MC Barriers
**Healthcare Provider Access**
Incompatible insurance
Lack of insurance coverage
Long wait time
No MC-authorized provider available
**Program Enrollment Process**
Difficulty navigating state website
Difficulty in knowledge of renewal requirements
Problems with renewal process
Problems with enrollment cost
**Medical Cannabis Accessibility**
Provider Stigma/Bias
No medical cannabis near them
Incompatible pharmacy hours
**Individual Factors**
Cost of MC products
Transportation issues
Caregiver responsibilities
Not safe to keep cannabis products in my home
Fear of discrimination at work
Lack of confidence to obtain MC products
Negative cultural perception of MC
Negative friend/family perception

*Note: Survey questions were posed to participants only if they answered “Yes” to “Did you ever have problems getting medicinal cannabis?”*

## Data Availability

Data can be made available upon reasonable request.

## Abbreviations

MC: Medical Cannabis
IMC: Illicit Medicinal Cannabis
OR: Odds Ratio
AOR: Adjusted Odds Ratio
SUBLIME: Study of Utahn’s Beliefs and Experiences with Integrative Medicine
DHHS: Utah Department of Health and Human Services
